# Sex-based differences in multidimensional clinical assessments of early-abstinence crack cocaine users

**DOI:** 10.1371/journal.pone.0218334

**Published:** 2019-06-21

**Authors:** Breno Sanvicente-Vieira, Diego Luiz Rovaris, Felipe Ornell, Anne Sordi, Leonardo Melo Rothmann, João Paulo Ottolia Niederauer, Jaqueline Bohrer Schuch, Lisia von Diemen, Felix Henrique Paim Kessler, Rodrigo Grassi-Oliveira

**Affiliations:** 1 Developmental Cognitive Neuroscience Lab, School of Health Sciences, Pontifícia Universidade Católica do Rio Grande do Sul, Porto Alegre, Rio Grande do Sul, Brazil; 2 Brain Institute of Rio Grande do Sul, Porto Alegre, Pontifícia Universidade Católica do Rio Grande do Sul, Rio Grande do Sul, Brazil; 3 Department of Psychiatry, Faculdade de Medicina, Universidade Federal do Rio Grande do Sul, Rio Grande do Sul, Porto Alegre, Brazil; 4 Attention Deficit Disorder Outpatient Program, Hospital de Clínicas de Porto Alegre, Rio Grande do Sul, Porto Alegre, Brazil; 5 Center for Drug and Alcohol Research, Hospital de Clínicas de Porto Alegre, Universidade Federal do Rio Grande do Sul, Porto Alegre, Brazil; 6 Laboratory of Immunosenescence, Graduate Program in Biomedical Gerontology, Pontifícia Universidade Católica do Rio Grande do Sul, Rio Grande do Sul, Porto Alegre, Brazil; University of Sao Paulo Medical School, BRAZIL

## Abstract

Crack cocaine use disorder (CUD) has been related to sex differences. This work aimed to compare the severity of drug use and the severity of other negative related outcomes in males and females with CUD. A total of 1344 inpatients (798 males and 546 females) with crack cocaine use disorder (CUD) were evaluated by a detailed multidimensional clinical assessment, including addiction severity and trauma exposure. Linear regression predicted higher drug use severity (*β* = 0.273, p < 0.001) and more problems in domains related to childcare issues (*β* = 0.321), criminal involvement (*β* = 0.108), work-related problems (*β* = 0.281) and social support impairments (*β* = 0.142) for females, all with p < 0.001. Alcohol problems were predicted to be higher in males (*β* = -0.206, P < 0.001). Females had higher rates of other mental disorders, particularly trauma and stress-related disorders (OR: 3.206, CI: 2.22, 4.61). Important sex differences also emerged in trauma history and HIV infection prevalence. CUD has a more severe clinical presentation among females facing early abstinence. Sex differences in the CUD course indicate the need for consideration of sex-specific interventions and research.

## Introduction

Cocaine use disorder is a chronic relapsing disorder characterized by drug-seeking behaviors, such as lack of control, social malfunctioning and risky drug use [1]. The disorder’s severity is determined by the number and extent of social and behavioral issues. When cocaine is smoked, i.e., consumed as crack cocaine, the withdrawal, effects and prognosis are considered worse than when cocaine is inhaled [2]. However, the etiology, pathophysiology, disorder course and treatment responses vary based on sex differences [3–5].

An estimated 18.8 million users use cocaine annually [6]. Although cocaine use is three times more prevalent among males [7], females have an increased risk of early onset of crack cocaine consumption [8, 9], and they have a faster progression from initial drug use to addiction (i.e., substance use disorder, SUD), an effect called “telescoping” [10]. Taking into account characteristics that contribute to the severity of crack cocaine use disorder (CUD), females have more social problems, and males face more problems with the law [11]. Additionally, females have higher rates of HIV [12] and comorbid psychiatric disorders, but personality disorders are more prevalent in males [13].

Such differences may arise from multiple biopsychosocial factors, including hormones, cultural shaping and emotional vulnerability, for example. However, mechanisms and outcomes are not well-documented [3]. From an environmental perspective, vulnerability to crack cocaine is suggested to have sex-based specificities because social differences are related to gender roles that males and females typically assume. Cultural determination can turn drug use into a transgressive or desired behavior due to social shaping, for instance. Moreover, across most historical periods and in various countries, drug use has been less acceptable for women than for men [14]. Moreover, most illegal drugs are only available in dangerous areas, which probably makes females more frequently avoid an initial search for the drug due to the possibility of violence [15].

On the other hand, after initial drug use, females in drug-dealing areas may become more vulnerable to victimization [16]. Along with social influences, evidence shows that gonadal hormones modulate cocaine-induced outcomes. The effects of the menstrual cycle indicate that cocaine-induced subjective “high” feelings are more pronounced in the follicular phase than in the luteal phase, particularly when cocaine is smoked [17]. Animal studies show that estrogen boosts cocaine-induced rewarding effects, but progesterone seems to reduce its effects [18]. The reward-seeking sensations associated with higher progesterone levels would contribute to neuroadaptations, leading to tolerance and abstinence [19].

Moreover, from a psychological point of view, recognized risk factors have different effects in males and females. Childhood maltreatment, highly prevalent among cocaine users [20], is a predictive risk factor for addiction [21]; however, evidence shows that this effect could be true for females but not males [22, 23]. Moreover, such negative events seems to worsen withdrawal and depressive symptoms in females [24].

Therefore, sex-based effects in several clinical dimensions may interact with each other and negatively influence various aspects in the lives of crack cocaine users. To our knowledge, no previous study with a large sample has focused on crack cocaine users and evaluated CUD severity considering various psychosocial dimensions. However, some work tested sex differences in variables that are indexes of CUD severity. For example, a previous study with 816 crack cocaine users in Brazil found that females have more financial, educational and childcare problems. Moreover, females showed higher HIV prevalence rates and more common sexual abuse history [11]. Similarly, other Brazilian study with 919 crack cocaine users revealed that males report higher lifetime polydrug use, while females have a higher prevalence of syphilis and report to consume higher crack cocaine amounts, and to had more often positive history of prostitution and sexual victimization [25]. In addition, a study with 227 cocaine users revealed that females have higher rates of bipolar disorder, posttraumatic stress disorder and psychotic-induced disorders [26].

### Aims of the study

Our main aim was to compare the severity of drug use and other negative outcomes related to cocaine use disorder (CUD) between males and females who use crack cocaine during early-abstinence. Additionally, we intended to test the effects of sex differences on rates of mental disorders, trauma exposure, drug use characteristics, and other negative CUD-related issues. Their identification in these life dimensions is important for determining better preventive and therapeutic strategies.

## Material and methods

### Design

We designed an observational cross-sectional study to investigate sex differences in inpatient crack cocaine users undergoing detoxification treatment in accordance with STROBE (Strengthening the reporting of Observational Studies in Epidemiology) [27].

### Participants

The total sample included 1344 participants (797 males). All participants were recruited from two public detoxification inpatient programs funded by the Brazilian government in Porto Alegre, Southern Brazil. Each unit was designed to receive males or females exclusively. Eligibility for the study included (a) being voluntarily hospitalized for detoxification of crack cocaine, (b) self-reporting recent use of crack cocaine before the hospitalization to ensure we were testing participants in early abstinence, (c) fulfilling DSM-IV criteria for CUD, (d) self-reporting crack cocaine as the primary drug of choice, (e) being 18 years old or older, and (f) having no cognitive deficits compromising the ability to answer the protocol. Cognitive deficits were defined by mental status exam. Clinical psychiatrists performed the inclusion/exclusion processes, including the clinical mental status exam.

Hospitalization for drug detoxification is one of the treatment options available in the public health-care system in Brazil. The Unified Health System (SUS, *Sistema Único de Saúde*, in Portuguese) guarantees universal and unrestricted access for all citizens to health care, including mental health [28]. Primary health care is the first and most universal level of this structure, aiming to provide comprehensive health care, promote preventive strategies and evaluate the complexity of each case, referring patients to specialized hospital care when necessary. Primary care is intended to employ a multidisciplinary team and to work in the community and in basic health units, which are standard caring centers for the community. Primary care provides access to more complex levels of the health-care system [29].

A patient diagnosed with a more complex condition, such as a severe SUD, will be referred to secondary or tertiary care. Tertiary care in the SUS comprises high-complexity conditions, which are mostly addressed in general hospitals focused on intensive care. For drug addiction, tertiary care occurs in psychiatric inpatient facilities, such as those where this study took place, or specialized wards [29]. Some non-governmental initiatives also offer treatment in therapeutic communities, but protocols and practices are not standardized [29]. Common symptoms that indicate the consideration of hospitalization are acute intoxication, withdrawal symptoms, psychiatric comorbidities, suicide ideation, aggressive behavior and psychotic symptoms [30]. It is also worth mentioning that people only receive primary, secondary or tertiary health care in the SUS if they admit themselves of their own will, as is mandated by law, with few exceptions. Therefore, hospitalization requires two criteria: (1) a referral from a primary or secondary service requiring hospitalization and (2) patient approval to receive the treatment. Before hospitalization, patients sign a form that includes their consent to the treatment and the referral from a health-care professional. In our study, only voluntarily hospitalized patients were included. Therefore, the included participants could have sought treatment in second-care services, therapeutic communities or specialized wards for acute symptomatic treatment. They probably decided on hospitalization due to the benefits of continuous treatment from an integrated multidisciplinary team and their intention to remain abstinent after detoxification.

At both psychiatric facilities where this study occurred, inpatients were kept in an abstinence-controlled medical unit and followed a standardized treatment protocol for up to three weeks voluntarily. During treatment, patients underwent a full health evaluation and followed a routine that included a schedule for hygiene, leisure activities, physical education, occupational activities, visits and group and individualized therapeutic psychosocial activities [30]. Most therapeutic activities were meant to motivate inpatients to seek secondary-care services after hospitalization. Participants also followed a planned diet and had a prescribed symptomatic cocaine detoxification protocol with neuroleptics, antidepressants and mood stabilizers as described previously [31].

The minimum sample size was estimated considering the mean and standard deviation of the ASI-6 drug severity composite scores from males and females with SUD assessed in a previous study [32]. Therefore, considering an alpha of 5% and beta of 80%, the minimum sample size for each group was 393 participants.

### Procedures

Data was extracted from March 2011 to December 2015. The invitation for research participation occurred on the 5^th^ day. Earlier in the hospitalization, most patients are subject to have acute drug intoxication or to feel extremely vulnerable, which can impact the ability of give consent to participate in the study [33]. If patients had no ability to understand the study objectives or showed acute symptoms, they were invited to another opportunity because their written informed consent was required. All measures occurred during the second week of detoxification to avoid acute interference of symptoms in the evaluation.

The research protocol was independent from the treatment, meaning that refusal to participate or exclusion had no influence on the standard treatment the facility carried out. Moreover, throughout the research protocol, participants were reminded that their participation was voluntary, they could refuse to participate and they could withdraw their consent. All the research protocol was reviewed and approved by the ethical committees of the institutions involved in this study (see below).

### Ethics

This study was conducted according the principles in the Declaration of Helsinki. All procedures were reviewed and approved by the Ethical Committee of Pontifícia Universidade Católica do Rio Grande do Sul (registration number: CEP 10/05214, November 2010) and the Ethical Committee of Hospital de Clínicas de Porto Alegre (registration number: 100002, January 2010). All participants provided written informed consent prior to any procedure in the study.

### Measures

The assessment protocol evaluated mental disorders, severity of substance use disorders, clinical and psychosocial characteristics and history of childhood trauma. In addition, we assessed other medical conditions, legal and labor issues, social support problems and family care issues. Participants were interviewed with the Structured Clinical Interview (SCID-I) [34] to identify mental disorders and confirm the CUD diagnosis. The substance use and abuse disorders module of the SCID-I were modified for this study. We considered both disorders (substance abuse and substance dependence) a single disorder, SUD, fitting DSM-5 criteria [35]. We also organized the disorders according to the DSM-5’s structure (e.g., considering trauma and/or stress-related disorders separate from anxiety disorders). We assessed lifetime and current (last 12 months) diagnoses. Because our study focused on crack cocaine users, we proposed a subdivision in which snorted cocaine was considered separately from smoked cocaine. Therefore, although the entire sample was formally diagnosed with stimulant (cocaine) use disorder, we evaluated current or lifetime snorted cocaine use disorder, crack cocaine use disorder and other stimulant use disorder.

Drug use characteristics, addiction severity and problems in other areas of psychosocial functioning were assessed with the Addiction Severity Index 6 (ASI-6) [36, 37], a structured interview that allows investigators to assess a range of domains often affected by alcohol and drug use. We used a validated Brazilian-Portuguese version [38]. The ASI-6 includes detailed information, including patterns of drug use, history of trauma and other life issues. Moreover, ASI-6 allows the computation of composite scores for the severity of nine domains that may be problematic in addiction: drug use, family-related problems, alcohol consumption, psychiatric issues, medical problems, legal issues, financial problems, lack of social support and social problems. The ASI-6 is one of the most used instruments for assessing the severity of drug users, being a valid and reliable measure [36, 37]. The composite scores are generated by considering different issues assessed by the interview. For example, for alcohol severity, it considers time of use, time since last use, problems because of alcohol use, need for special attention to such problems and the frequency of all these occurrences. The composite scores are continuous variables, with higher scores indicating more severe negative impacts in each domain. Although it has been pointed the relevance of crating cutoffs for ASI-6 composite scores, there is no data supporting it [39]. It is worth saying that we made a single adaptation to the scoring system: we considered prostitution non-formal work in accordance with Brazilian law instead of considering it a crime as the original instrument does.

We assessed childhood trauma with the Brazilian version of the Childhood Trauma Questionnaire [40]. The CTQ is a 5-point Likert-type scale with 28 items that assesses how often abuse or neglect occurred when the participants were children. The CTQ allows for the assessment of the severity of various types of childhood maltreatment (emotional, physical and sexual abuse; emotional and physical neglect) [41]. Traumatic events that occurred during adulthood are also an important variable because violence is one of the most common causes of death among crack cocaine users in Brazil [42]. The ASI-6 includes specific questions regarding type of and age at traumatic experiences.

Some data were missing from our study. Not all participants had available data for all measures because during assessment, research protocols changed. Therefore, participants assessed before 2013 had no CTQ assessment, and those assessed before 2010 had no SCID-I assessment. Moreover, some participants lacked details for the ASI-6.

### Analyses

We computed descriptive analyses (i.e., mean, standard deviation, number of observations and percentages) considering the whole sample. We used chi-square tests with the Yates correction for continuity for categorical (binary) variables or Fisher’s test when the number of expected observations was small. We also calculated the odds ratio (OR) for each psychiatric disorder (current and lifetime) and its 95% confidence intervals (CIs). We coded males as 1 (reference category) and females as 2. For continuous variables, we used Student’s t-test or Mann-Whitney when appropriate. We included Cohen’s *d* effect sizes for parametric tests and estimated *r* for the non-parametric ones. To account for multiple comparisons, we corrected all *P*-values for multiple testing with a false discovery rate (FDR) test [43], assuming a corrected *P*-value of 0.05 or lower.

After completing the above steps, we focused in testing if sex differences indeed influence the severity of problems in different life domains of crack cocaine users, even adjusting it for biasing factors. Because we investigated a number of characteristics that potentially contribute for negative outcomes, we tested if sex would still have a relation with severity outcomes, despite multiple confounding and biasing variables. For this purpose, we considered the ASI-6 composite scores as severity measures, since these are valid and reliable measures for measuring negative life outcomes in different life domains [36, 37]. We tested ASI-6 composite scores as dependent variables in linear regression models with the backward method manually. We included in the models all variables associated with sex (the study factor) and ASI-6 scores (outcomes). Because there was a high amount of collinearity among some variables, we always selected the variable that most encompassed the phenomenon. For example, if significant differences emerged between actual and lifetime specific mental disorders, we selected the lifetime one for inclusion in the model. We repeated the analysis with all variables included in each model, removing from the next run the least significant variable until the regression coefficient reached the highest value and the model kept only significant variables and sex as predictors. Then we considered the β value the adjusted value for sex in the relation with each negative outcome. Moreover, we also checked if the remaining model was statistically significant and we reported all variables that remained as more predictive than sex within the linear model.

## Results

### Sociodemographic characteristics

The mean age of the sample was 32.66 (*SD* = 10.44) years. Males had a mean age of 32.4 (*SD* = 11.57) years old, in comparison to 33.04 (*SD* = 8.53) years old from females. Groups did not show difference in the mean age (*t* = -1.172, *P* = 0.241), and when we divided the age in categorical groups by the age range, it did also not show any difference. The overall monthly income was 315.86 (*SD* = 423.88) dollars. There was significant difference (*U* = -6.750, *P* <0.001) in the comparison for income, males (*M* = 355.65, *SD* = 413.18) had higher incomes in dollars than females did (*M* = 257.87, *SD* = 432.88). When income was divided in Brazilian income classification [44], most of the sample was classified as C, D or E, meaning medium-low, low and most low classes. Differences indicated higher proportions of males in the B classes in comparison to females, and significant higher proportion of females in classes C than males. Number of children (for all sample, *M* = 1.76, *SD* = 1.95) showed differences (*U* = -9.951 2, *P* <0.001), with females having more children than males did (*M* = 1.37, *SD* = 1.71).

Other sample characteristics are shown in Table 1. Most of the sample self-declared as white in both groups, but when we tested differences in proportions, a higher proportion of males self-declared as white, and more females self-declared as black. Most of the sample was single. When comparing proportions of partner status, it was noticed that females had stable relationships more often than males. Males, on the other hand, reported higher proportions of divorce.

**Table 1:** Sociodemographic characteristics.

	All (n = 1344)	Males (n = 797)	Females (n = 547)	Statistics	
	*n (%)*	*n (%)*	*n (%)*	*X*^*2*^	*P*
**Age range**					
Under 20 years old	133 (9.9)	84 (10.5)	49 (9)	0.741 ^1^	0.389
21–30 years old	444 (33)	261 (32.7)	183 (33.5)	0.045 ^1^	0.832
31–40 years old	494 (36.8)	278 (34.9)	216 (39.5)	2.767 ^1^	0.096
41–50 years old	203 (15.1)	128 (16.1)	75 (13.7)	1.219 ^1^	0.270
More than 51 years old	70 (5.2)	46 (5.8)	24 (4.4)	0.994 ^1^	0.319
**Income classification based on earnings per month** ^2^					
A1 and A2	9 (0.7)	5 (0.6)	4 (0.7)		0.535 ^3^
B1 and B2	146 (10.9)	114 (14.3)	32 (5.9)	23.074 ^1^	<0.001
C1 and C2	659 (49)	372 (46.7)	287 (52.5)	4.127 ^1^	0.042
D and E	530 (39.4)	306 (38.4)	224 (41)	0.784 ^1^	0.376
**Ethnicity**					
White	640 (47.6)	418 (52.4)	222 (40.6)	17.825 ^1^	<0.001
Black	384 (28.6)	192 (24.1)	192 (35.1)	18.732 ^1^	<0.001
Other	319 (23.7)	187 (23.5)	133 (24.3)	0.087 ^1^	0.768
**Partner status**					
Single (never married)	809 (60.2)	482 (60.5)	327 (59.8)	0.040 ^1^	0.842
Married/ Living together	418 (31.1)	227 (28.5)	191 (34.9)	5.973 ^1^	0.015
Widowed	61 (4.5)	44 (5.5)	17 (3.1)	3.819 ^1^	0.051
Divorced/Separated	56 (4.2)	44 (5.5)	12 (2.2)	8.177 ^1^	0.004
**Education level** ^4^					
Basic (1–8 years of study)	875 (65.1)	490 (61.5)	385 (70.4)	10.930 ^1^	0.001
Intermediate (9–13 years of study)	423 (31.5)	268 (33.6)	155 (28.3)	3.967 ^1^	0.046
High (more than 14 years of study)	46 (3.4)	39 (4.9)	7 (1.3)	11.745 ^1^	0.001

^1^ Chi-Square Continuity Correction.

^2^ Income classification was based in the Brazilian Criteria [44], which has 8 classes (A1, A2, B1, B2, C1, C2, D and E). For making easier the representation, we show classes 1 and 2 together, likewise D and E.

^3^ Fisher’s exact test.

^4^ Categories were based in the Brazilian educational stages.

### Drug use characteristics

Table 2 shows comparisons in substance use characteristics for males and females. A higher proportions of males than of females reported having used alcohol, sedatives, hallucinogens, stimulants other than cocaine or heroin. The last two substances had results that did not remain significant after multiple corrections. The only drug that females reported to have used more frequently than males was tobacco. For age of first use, males reported first use of alcohol at a younger age than females did; females reported a younger age of first use of tobacco and crack cocaine. Only the last showed significance at moderate effect size. Males reported more years of use of alcohol, cannabis and stimulants other than cocaine/crack cocaine. Females reported more years of use of tobacco and opiates other than heroin. Females had more detoxification hospitalizations and a younger age of first drug use treatment.

**Table 2:** Comparisons in drug use characteristics and treatment related indexes between males and females.

	All (n = 1344)	Males (n = 798)	Females (n = 546)	Statistics			
	*M (SD)*	*M (SD)*	*M (SD)*	*t/U*	*P*	Corrected *P*	Effect size/ OR (95% CI)
**Crack cocaine**							
Use in life (n, %)	1344 (100)	798 (100)	546 (100)				
Age of first use	22.21 (7.7)	24.21 (7.7)	19.29 (6.8)	12.229 ^1^	<0.001	<0.001	0.667
Years of use	7.89 (5.62)	8.0(5.8)	7.6 (5.3)	1.384 ^1^	0.167	0.437	0.075
**Alcohol**							
Use in life (n, %)	1314 (97.8)	788 (98.9)	526 (96.2)	9.706 ^2^	0.002	0.007	0.28 (0.13, 0.62)
Age of first	14.97 (3.9)	14.6 (10.4)	16.3 (8.7)	-2.553 ^3^	0.011	0.03	-0.070
Years of use	7.0 (9.9)	9.4 (11.2)	3.4 (6.2)	-10.567 ^3^	<0.001	<0.001	-0.291
**Tobacco**							
Use in life (n, %)	1167 (86.8)	672 (84.3)	495 (90.5)	10.291 ^2^	0.001	0.003	1.77 (1.25, 2.49)
Age of first use	14.1 (4.8)	14.4 (4.7)	13.7 (4.8)	-4.006 ^3^	<0.001	<0.001	-0.117
Years of use	15.3 (9.8)	14.6 (8.7)	16.3 (8.7)	-2.931 ^1^	0.003	0.01	- 0.171
**Cannabis**							
Use in life (n, %)	1197 (89.1)	703 (88.2)	494 (90.3)	1.127 ^2^	0.260	0.647	1.24 (0.87, 1.77)
Age of first use	15.3 (4.9)	15.41 (5.3)	15.3 (4.3)	-0.646 ^3^	0.519	0.999	-0.018
Years of use	8.0 (8.5)	8.5 (8.9)	7.3 (7.7)	-2.120 ^3^	0.034	0.104	-0.061
**Powder cocaine**							
Use in life (n, %)	1141 (84.9)	683 (85.7)	458 (83.7)	0.831 ^2^	0.362	0.874	0.85 (0.63, 1.16)
Age of first use	18.4 (5.8)	18.4 (5.6)	18.5 (5.9)	-0.161 ^3^	0.872	0.999	-0.004
Years of use	6.8 (7.1)	6.9 (7.6)	6.7 (6.3)	-1.081 ^3^	0.280	0.686	-0.032
**Other stimulants**							
Use in life (n, %)	145 (10.8)	98 (12.3)	47 (8.6)	4.246 ^2^	0.039	0.117	0.67 (0.46, 96)
Age of first use	21.4 (7.9)	21.8 (8.3)	20.7 (7.1)	-0.313 ^3^	0.754	0.999	-0.025
Years of use	5.6 (5.2)	6.3 (5.7)	3.9 (3.8)	-3.088 ^3^	0.002	0.007	-0.256
**Sedatives**							
Use in life (n, %)	390 (29)	258 (32.4)	132 (24.1)	10.29 ^2^	0.001	0.003	0.66 (0.52, 0.85)
Age of first use	26.2 (10.5)	26.2 (10.3)	26.3 (10.8)	-0.127 ^1^	0.899	0.999	-0.012
Years of use	3.0 (5.6)	3.1 (6.1)	2.8 (4.5)	-0.772 ^3^	0.440		-0.039
**Hallucinogens**							
Use in life (n, %)	194 (14.4)	141 (17.7)	53 (9.7)	16.176 ^2^	<0.001	<0.001	0.49 (0.35, 0.69)
Age of first use	21.0 (7.9)	21.29 (6.2)	20.13 (5.4)	1.192 ^1^	0.235	0.597	0.183
Years of use	0.7 (2.4)	0.9 (2.8)	0.3 (1.0)	-1.543 ^3^	0.123	0.332	-0.110
**Inhalants**							
Use in life (n, %)	545 (40.6)	325 (40.8)	220 (40.2)	0.022 ^2^	0.882	0.999	0.97 (0.78, 1.22)
Age of first use	16.9 (6.1)	16.1 (4.8)	17.8 (7.3)	-1.630 ^3^	0.103	0.283	-0.069
Years of use	1.6 (3.8)	1.6 (4.0)	1.6 (3.4)	-1.532 ^3^	0.125	0.332	-0.065
**Heroin**							
Use in life (n, %)	35 (2.5)	27 (3.4)	8 (1.5)	4.011 ^2^	0.045	0.133	0.42 (0.19, 0.93)
Age of first use	21.0 (7.9)	15.41 (5.3)	15.3 (4.3)	-0.868 ^3^	0.385	0.915	-0.146
Years of use	0.6 (1.3)	8.5 (8.9)	7.3 (7.7)	-0.192 ^3^	0.848	0.999	-0.032
**Opiates other than heroin**							
Use in life (n, %)	76 (5.7)	60 (7.5)	16 (2.9)	12.035 ^2^	< 0.001	<0.001	0.37 (0.21, 0.65)
Age of first use	26.7 (10.2)	26.8 (11.1)	26.1 (6.1)	0.240 ^1^	0.811	0.999	0.055
Years of use	2.6 (1.8)	1.9 (0.9)	5.0 (2.3)	-5.209 ^3^	<0.001	<0.001	-0.597
**Treatment indexes**							
Hospitalizations for drug use	4.4 (6.1)	4.4 (6.6)	4.22 (5.2)	-1.724 ^3^	0.085	0.238	-0.047
Detoxifications	2.9 (4.5)	2.4 (4.6)	3.7 (4.3)	-11.360 ^3^	<0.001	<0.001	-0.309
Age of first drug use treatment	26.0 (8.3)	26.9 (8.5)	24.6 (7.8)	5.188 ^1^	<0.001	<0.001	0.283

For age of first use and years of use of each drug, means were calculated considering those participants who had reported previous use of each substance in life. In the case of t-tests, effect size reported refers to Cohen’s *d*. For chi-square, instead of effect sizes, the odds ratio is reported, and in cases of Mann-Whitney, to *r* equivalent to *d*.

^1^ Mann-Whitney Standardized Z.

^2^ t-test value.

^3^ Pearson chi-square.

### Negative life issues

Sex differences and comparisons in life issues are shown in Table 3. Regarding housing, females had been homeless more often. Medical issues, HIV and other medical conditions were also more prevalent among females. Moreover, females had non-formal occupations more frequently than males. Among females, the most common non-formal work was prostitution: 18.6% reported having worked in prostitution (not including cases in which they had exchanged sex for drugs), and 17.5% reported other non-formal occupations. Among males, 2.3% reported having worked in prostitution. Regarding legal problems, males had a higher proportion of previous arrests. In social life, females reported having fewer close friends and less contact with close friends and siblings in the last 30 days. At the same time, males reported having less contact with a partner in the last 30 days. Moreover, in terms of interpersonal conflicts with close people, males reported a higher frequency of fights or arguments in the last 30 days than females did. History of suicide also showed sex-based differences: females more often had a history of suicide attempts, either any time in the life course or in the last 30 days.

**Table 3:** Comparisons of negative life outcomes associated with CUD between male and female crack cocaine users.

	All (n = 1268)	Males (n = 748)	Females (n = 520)	Statistics			
	*n (%)*	*n (%)*	*n (%)*	*X*^*2*^*/ t/Z*	*P*	*Corrected P*	*Effect size/ OR (CI)*
**Housing issues**							
Homeless in lifetime	569 (44.9)	290 (38.8)	279 (53.7)	26.872 ^1^	<0.001	<0.001	1.82 (1.45, 2.29)
Homeless in last 6 months	419 (33)	200 (26.7)	219 (42.1)	32.093 ^1^	<0.001	<0.001	1.99 (1.57, 2.52)
Homeless in last 30 days	315 (24.8)	133 (17.8)	182 (35)	47.796 ^1^	<0.001	<0.001	2.49 (1.92, 3.22)
**Medical Issues**							
HIV	150 (11.8)	45 (6)	105 (20.2)	57.752 ^1^	<0.001	<0.001	3.95 (2.73, 5.72)
Medical Condition Other than HIV	719 (56.7)	400 (53.5)	319 (61.3)	7.422 ^1^	0.006	0.02	1.38 (1.10, 1.73)
**Employment issues**							
Unemployment	597 (47.1)	345 (46.1)	252 (48.5)	0.583 ^1^	0.445	0.999	1.09 (0.87, 1.37)
Not formal employment	267 (21.1)	102 (13.6)	165 (31.7)	59.335 ^1^	<0.001	<0.001	2.94 (2.22, 3.89)
**Legal Problems**							
Already Arrested	414 (32.6)	286 (38.2)	128 (24.6)	25.261 ^1^	<0.001	<0.001	0.52 (0.41, 0.67)
**Social life**							
Number of close friends—M (SD)	1.77 (0.9)	2.01 (3.33)	1.35 (3.1)	-4.280 ^2^	<0.001	<0.001	-0.139
Interactions in last month							
In touch with a partner	735 (58)	400 (53.5)	335 (64.4)	14.641 ^1^	<0.001	<0.001	1.57 (1.25, 1.98)
In touch with a sibling	941 (74.2)	585 (78.2)	356 (68.5)	14.723 ^1^	<0.001	<0.001	0.60 (0.46, 7.77)
In touch with close friends	486 (38.4)	311 (41.6)	175 (33.7)	7.779 ^1^	0.005	0.014	0.71 (0.56, 0.90)
Fight with close people	865 (68.2)	546 (73)	319 (61.3)	18.664 ^1^	<0.001	<0.001	0.58 (0.46, 0.74)
**Suicide**							
Suicide attempt in life	524 (41.3)	259 (34.6)	265 (51)	33.090 ^1^	<0.001	<0.001	1.96 (1.56, 2.46)
Suicide attempt in last 30 days	209 (16.5)	81 (10.8)	128 (24.6)	41.358 ^1^	<0.001	<0.001	2.68 (1.56, 2.46)
**Trauma History**							
Physical harassment	760 (59.6)	497 (66.4)	263 (50.6)	31.504 ^1^	<0.001	<0.001	0.51 (0.41, 0.65)
Witnessed a hard crime	862 (68)	548 (73.3)	314 (60.4)	2.782 ^1^	<0.001	<0.001	0.55 (0.43, 0.70)
Sexual harassment	304 (24)	70 (9.4)	234 (45)	211.841 ^1^	<0.001	<0.001	7.92 (5.86, 10.70)
Raped as an adult	185 (14.6)	50 (6.7)	135 (26)	89.935 ^1^	<0.001	<0.001	4.89 (3.45, 6.92)
**Childhood Trauma**	*Mean* (SD)	*Mean* (SD)	*Mean* (SD)				
Total CTQ score	48.8 (18.1)	47.6 (16.3)	50 (19.6)	-2.119 ^3^	0.034	0.104	-0.137
Physical Neglect	8.8 (3.8)	8.8 (3.6)	8.8 (4.0)	-0.837 ^2^	0.403	0.944	-0.026
Emotional Neglect	11.0 (5.3)	10.4 (4.7)	11.5 (5.8)	-3.412 ^3^	0.001	0.003	-0.221
Sexual Abuse	7.2 (4.4)	6.5 (3.5)	8.0 (4.9)	-5.408 ^2^	<0.001	<0.001	-0.173
Physical Abuse	10.0 (5.3)	10.3 (5.3)	9.6 (5.2)	-2.445 ^2^	0.014	0.045	-0.078
Emotional Abuse	11.7 (5.4)	11.5 (5.2)	11.9 (5.6)	-1.185 ^2^	0.236	0.597	-0.076

In the case of t-tests, reported effect size refers to Cohen’s *d*, and in cases of Mann-Whitney, to *r* equivalent to *d*. For categorical variables where chi-square or Fisher’s exact test were used, the OR is reported. For number of close friends and CTQ total score and scores for each subscale, data are shown in mean and standard deviation. For childhood trauma, data included was restricted to those participants who had answered the CTQ, 473 males and 499 females.

^1^ chi-square

^2^ Mann-Whitney standardized Z

^3^ t-test value

#### Trauma

Descriptive data for childhood and adult history of traumas are presented in Table 3. Sex-based differences indicated that in adulthood, males more often suffered physical harassment and witnessed hard crimes. Females more often suffered sexual crimes, reporting higher rates of sexual harassment and rape during adulthood. Among those participants who suffered some type of sexual crime, 77.6% also experienced sexual aggression during childhood.

The CTQ, used to compare the severity of childhood maltreatment, was answered by 972 participants (473 males and 499 females). Using the CTQ continuous scores, sex-based differences indicated males had more intense histories of physical abuse, and females had higher scores for total CTQ, emotional neglect and sexual abuse.

### Psychiatric comorbidities

**Fig 1:**
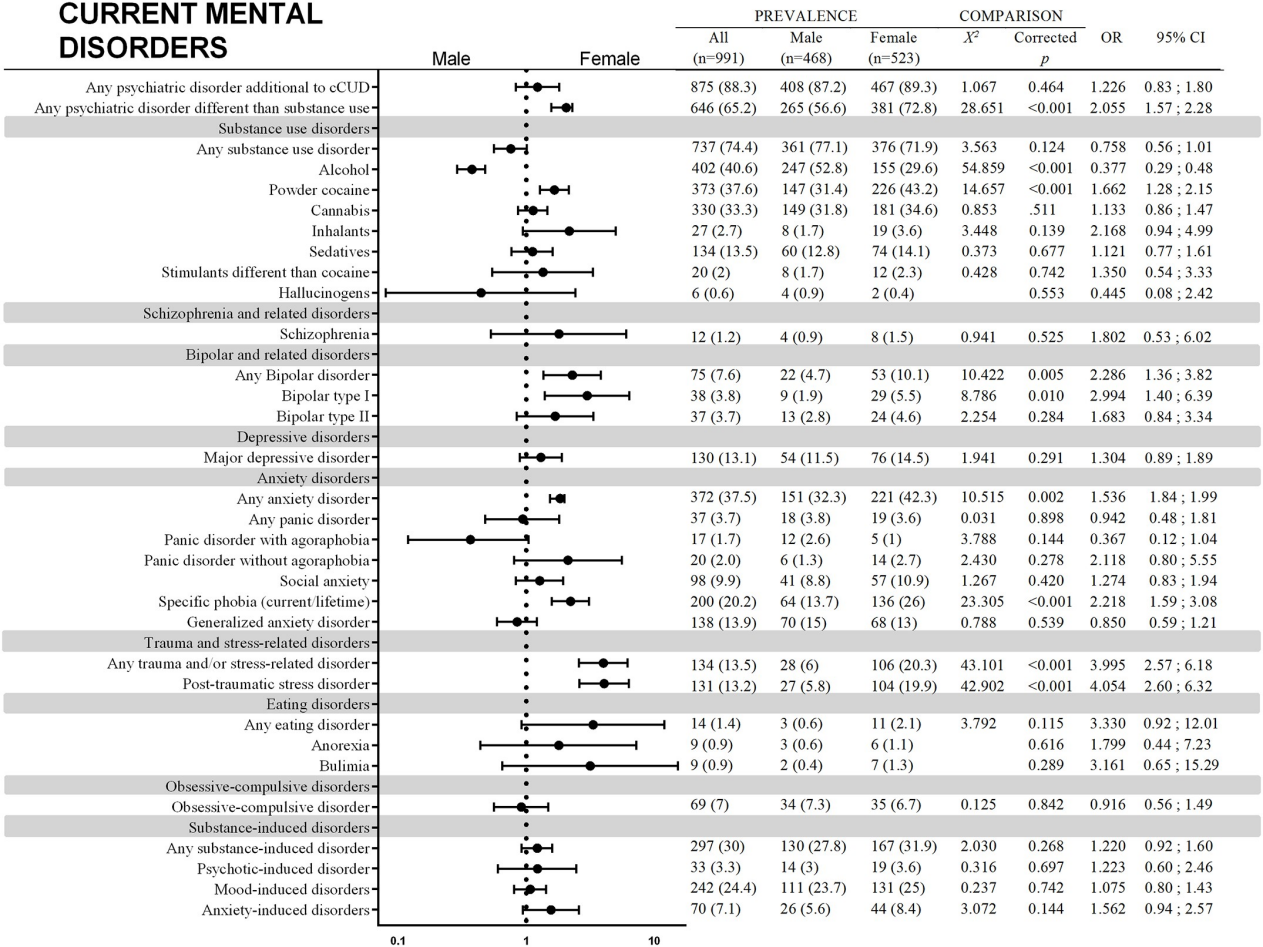
Comparisons of sex differences in current mental disorders among crack cocaine users hospitalized for detoxification. For comparisons without shown value for X^2^, comparisons were run with Fisher’s exact test. The farther left the point is located, the higher the risk is of that condition among males. The farther right the point is located, the higher the risk is of that condition among females.

**Fig 2:**
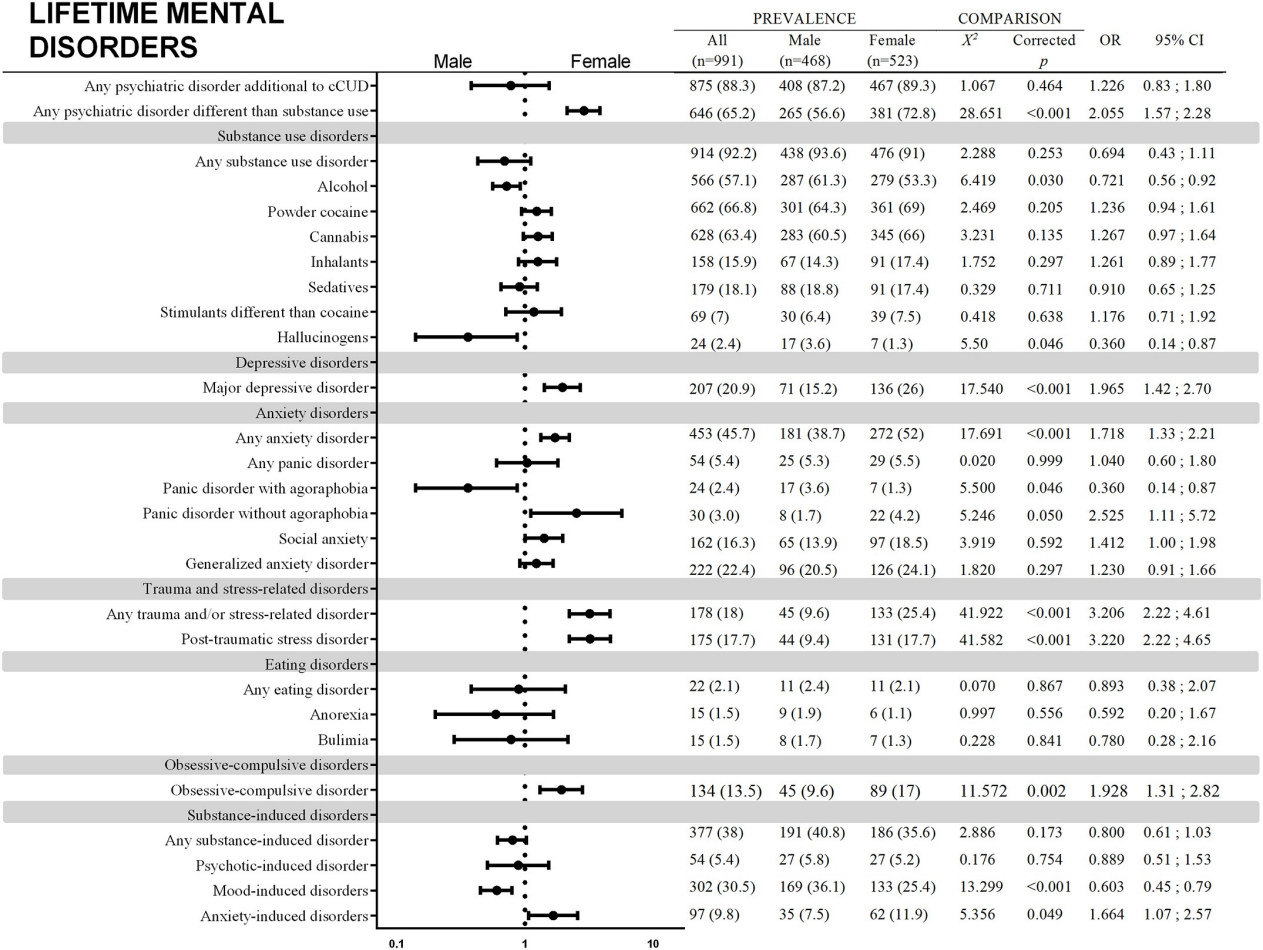
Comparisons of sex differences in lifetime mental disorders among crack cocaine users hospitalized for detoxification. For comparisons without shown value for X^2^, comparisons were run with Fisher’s exact test. The farther left the point is located, the higher the risk is of that condition among males. The farther right the point is located, the higher the risk is of that condition among females.

A total of 991 participants answered the SCID-I. Figs 1 and 2 show comparisons and forest plots based on ORs and 95% CIs as well as prevalence for current and lifetime psychiatry comorbidities, respectively. Females had more mental disorders than males when substance use disorders were not taken into account. Comparing individual groups of current disorders, males had a higher prevalence only for alcohol use disorder. Females, on the other hand, had higher estimates for general trauma and/or stress-related disorder, post-traumatic stress disorder, general anxiety disorder and powder cocaine use disorder (not considering crack cocaine). More differences appeared if we considered lifetime comorbid disorders. Males showed more hallucinogen use disorder, panic with agoraphobia and mood-induced disorders. Females had more general trauma and/or stress-related disorder, post-traumatic stress disorder, anxiety disorder, major depressive disorder, obsessive-compulsive disorder and anxiety-induced disorders. Moreover, females had more specific phobias, general bipolar disorder and bipolar disorder type I.

#### Severity of CUD-related outcomes

Available data for ASI-6 score comparisons were obtained from the 1268 participants (748 males and 520 females) who answered the entire interview, making the composite score computation possible. We found significant sex differences in eight of the nine ASI-6 individual composite scores. Table 4 shows a comparison of ASI-6 scores. The results remained significant after corrections with at least moderate effect size were restricted to indications of overall drug use severity (ASI-6 Drugs score) and employment problems (ASI-6 employment problems score) in females. A significant difference with a large effect size indicated females also experienced more severe problems related to childcare (ASI-6 Childcare Problems score) than males. In addition, the data showed worse psychosocial functioning of females, particularly related to emotional suffering (ASI-6 Psychological Problems score), non-psychiatric conditions (ASI-6 medical problems score), justice issues (ASI-6 legal problems score) and social support problems (ASI-6 Social Support score). The data for males indicated more problems related to alcohol use severity (ASI-6 alcohol score).

**Table 4:** ASI-6 composite score comparisons and linear regression adjusted effects of sex.

ASI-6 Scores	All (n = 1268)	Males (n = 748)	Females (n = 520)	Statistics			
				Comparative results		Linear regression adjusted values for sex	
	M (SE)	M (SE)	M (SE)	Z	Corrected *P*	*β*	*P*
Drugs	52.0 (0.2)	50.3 (0.2)	54.6 (0.2)	-9.735	<0.001	0.273 ^1^	<0.001 **
Alcohol	50.7 (0.2)	52.4 (0.4)	48.3 (0.3)	-6.151	<0.001	-0.206 ^1^	<0.00l **
Childcare Problems	55.2 (0.2)	52.0 (0.2)	59.7 (0.4)	-13.851	<0.001	0.321 ^1^	<0.001 **
Psychological Problems	50.4 (0.2)	49.3 (0.3)	52.0 (0.3)	-7.196	<0.001	-0.044 ^2^	0.254 **
Medical Problems	49.1 (0.2)	48.1 (0.3)	50.5 (0.3)	-4.615	<0.001	0.022 ^3^	0.627 **
Legal Problems	51.2 (0.2)	50.3 (0.2)	52.6 (0.3)	-6.447	<0.001	0.108 ^4^	<0.001 **
Employment Problems	37.1 (0.1)	35.6 (0.2)	39.1 (0.1)	-10.755	<0.001	0.281 ^1^	<0.001 **
Social Support Problems	39.4 (0.2)	38.2 (0.3)	41.0 (0.3)	-5.411	<0.001	0.142 ^5^	<0.001 **
Social Problems	55.1 (0.2)	55.1 (0.3)	54.9 (0.3)	-0.460	0.999	-	-

Table shows the comparison of males and females on each ASI-6 composite score and the β value for sex in linear models that included all significant sex differences investigated, as a measure for the relationship between sex and the investigated ASI-6 composite score. The *P*-value for adjusted result refers to the significance level for sex in the adjusted model.

* indicates that the remaining model is significant at the last step of the linear regression, with *p <* 0.05.

** indicates that the remained model is significant at the last step of the linear regression, with *p <* 0.001.

^1^ Adjusted model remained only with sex.

^2^ Adjusted model remained with seven variables with stronger or equivalent prediction than sex: ASI-6 Drugs, Medical Problems, Alcohol and Children Problem Scores, along with lifetime Post-Traumatic Stress Disorder, sexual abuse CTQ score and suicide attempt.

^3^ Adjusted model remained with seven variables with stronger or equivalent prediction than sex: ASI-6 Alcohol, Psychiatric, Social Support Problems, Social Problems and Employment Scores, Physical Abuse CTQ score and having been homeless.

^4^ Adjusted model remained with three variables with stronger or equivalent prediction than sex: ASI-6 Drugs, Medical Problems, Alcohol and Childcare Problems Scores, besides lifetime Post-Traumatic Stress Disorder, sexual abuse CTQ score and suicide attempt.

^5^ Adjusted model remained with only one variable with stronger or equivalent prediction than sex: ASI-6 Medical Problems score.

### Adjusted results

We tested the predictive values of sex for each specific ASI-6 composite score, evaluating multidimensional severity by using linear regression models. Sex remained a significant predictor for ASI-6 Drugs, Children Problems, Employment Problems and Social Support composite scores, showing females were more impacted in these domains of psychosocial functioning. Sex also remained a significant predictor of the ASI-6 Alcohol composite score, indicating more severity related to drinking among males. Adjusted results, indicating sex *β* values in the remaining models, likewise the list of variables remained in each model are in Table 4.

## Discussion

In this study, we found sex differences in the severity of drug use and psychosocial problems across multiple clinical domains in a sample of crack cocaine users who were in early abstinence. Our findings reinforce that among those with CUD, males and females suffer from different consequences of drug use [8, 11, 12, 45, 46]. This work advances in comparison to a similar previous investigations on sex differences that assessed indexes of disease severity in crack cocaine users [11, 25], since we assessed a larger sample, but most importantly because we considered testing in multiple potentially impacted domains of life, using reliable and valid measures of it. Furthermore, we tested results on the severity of psychosocial problems considering the interference of comorbidities and problems in other areas of life, specially exposure to childhood maltreatment and lifetime victimization. Thus, we provided a more comprehensible and complete piece of data for supporting sex differences in crack cocaine use disorder. Results are relevant for targeting better interventions and identifying specific major problems in males and females who use crack cocaine to primarily address the most common issues. As secondary objectives, we compared males and females in domains other than drug use. Our findings support assumptions of sex-specific trajectories for drug use [19, 47]. Overall, females had a higher severity of problems related to childcare, criminal involvement, employment issues and social support problems, and males showed more severe alcohol-related problems. Additionally, we found females had more CUD-related negative outcomes, such as housing problems, prevalence of HIV and general mental disorders. Males more often had a history of alcohol use than females. These results may be considered to improve prevention strategies and policies; likewise, future directions in research also need to be acknowledged.

Some findings reflect issues that in fact occurred before any drug use, such as history of childhood trauma, which mostly happened before initial drug use. Therefore, some sex differences noticed here may have influenced the path to drug use disorder together with genetic factors in sex-specific manners and may have a bearing on future preventive strategies [19, 48]. Childhood maltreatment was shown to impact males and females differently [23]. Among crack cocaine users, females reported higher rates of sexual abuse and emotional neglect, and males reported more physical abuse. Although global estimates of childhood maltreatment are not conclusive about sex differences in the prevalence of such experiences [49], we have found data in line with our findings [50, 51]. These results stress that the relationship between early negative experiences and crack cocaine use still require better understanding, and sex-specific effects must be considered because females with such experiences receive more preventive care for drug use [22].

About the use of substances different from crack cocaine, estimates for lifetime use of each of these substances was higher than 80% in our sample. This finding matches with previous research about high rates of use of other substances for crack cocaine users [52]. Sex differences related to lifetime alcohol and tobacco use were also in line with evidence that male crack cocaine users have a higher history of polydrug use [25]. Sex differences in this regard were considerable for current alcohol use disorder, with males having higher prevalence. The lifetime use of such substances may have an impact in the liability for crack cocaine use, despite the existence of a SUD or not. For treatment strategies, alcohol use disorder may require a constant caution, particularly for males.

Moreover, differences emerged in lifetime prevalence of other mental disorders than SUDs. Although the current prevalence of comorbidities also showed differences, stronger results were shown in lifetime disorders. Indeed, we confirmed the hypothesis that comorbidities with CUD would be more common among females than males [9, 13]. The confirmation of this assumption makes the observation of comorbidities mandatory among female crack cocaine users. Special attention must be paid to trauma and/or stress-related disorders in female health services because this was one of our study’s major findings. In addition, it is worth stating that as most of the differences did not emerge in participants’ current status, it is possible to suggest partial confirmation of the self-medication hypothesis [53] for CUD development in females [54]. Such results combined with previous data point to the need for primary mental health care, especially among females, as a way to target the avoidance of problematic crack cocaine use later in life. Additionally, treatment programs for males and females should focus on co-occurrence of mental disorders, but if it is necessary to focus on specific disorders, SUDs are more common among males whereas among females, a wider range of disorders may be investigated.

Regarding sex-specific trajectories, various ages at first use were found for crack cocaine, which is consistent with early documentation from smaller samples of drug users [8, 9]. Because we found no differences in ages at first use for other substances and we found females had an increased severity of drug use, we believe our data circumstantially supports the “telescoping effect” [10] in crack cocaine users; however, it requires more investigation.

Considering issues that may be of critical importance due to the most negative outcomes associated with them, sex differences also appear in the two most reported common causes of death in crack cocaine users (i.e., HIV complications and violence victimization) [42]. Regarding violence, the difference is small, but males reported having suffered more physical assaults, and females reported having suffered more sexual harassment. In contrast, HIV infection is remarkably more common among females, as are other clinical disorders. These findings are in line with previous data [11, 12] and make clear the need for complete medical attention for users, particularly females.

Sex differences in inpatients for crack cocaine detoxification are widespread, which is clear when one takes into account the domains assessed by the ASI-6. Across nine domains of the scale, sex differences appeared in eight. Results remained significant at a considerable level even after corrections and adjustments were made for six of them. In accordance with these results, differences emerged in behaviors related to social engagement, risks, employment and drug use. All the differences reinforce current literature about existing widespread differences in crack cocaine/cocaine use [3, 47, 55–57]. Moreover, such data also show that appropriate interventions are also needed for males, probably targeting the avoidance of the use of other substances, particularly alcohol. For females, a more complex picture emerges due to problems in more areas of life. In addition, the age at which preventive strategies are used needs to be different for males and females because of differences in the age at first use.

Our findings need to be interpreted in light of some limitations. Generalizations of results are tentative because our data came from participants in a single city. Future multicenter studies can help in this sense. Considering the results, possible gender differences require attention as intervenient variables. Our work assumed a cisgender/heterosexual perspective, but due to sociocultural interferences and gender stereotyping, sexual orientation has an impact on psychological status [58], which we did not address here. Although trained psychologists and psychiatrists conducted the clinical assessments, the evaluations required participants’ collaboration and precision. Their psychological condition during hospitalization and even the unclear starting point of the symptoms make diagnosis difficult. Particularly, some diagnoses were very hard to define because the time of initial drug use was unknown. Clinical rounds were conducted to minimize such problems, but the nature of the phenomenon is clearly hard to define. Moreover, both facilities served primary drug users, meaning that drug users with more severe psychological problems in other domains probably would go to other facilities and were not investigated here.

Another point that requires care in the interpretation of our results is that we did not investigate street users, only users who were inpatients of detoxification units in specialized hospitals in Southern Brazil. This geographic region has a very high prevalence of crack cocaine use in Brazil. This region also contrasts with others because the majority of the population has European ancestry. This fact could partially explain why our sample has a profile slightly different from previous works with crack cocaine users in Brazil [7, 12]. Finally, previous research supports sex differences related to other drugs, thus we cannot exclude the possibility that our results could also be impacted by the withdrawal of other drugs, such as nicotine [59], alcohol [60], cannabis and other drugs [61], or even for the combination of all these substances, which may require further exploration.

These data are important evidence showing that CUD manifests in a sex-specific manner. Our work contributes documentation of sex differences among crack cocaine users with a larger sample than most previous studies, and multiple clinical domains were assessed at once. Moreover, we investigated crack cocaine users that are probably at greater risk of more severe symptoms because they sought detoxification treatment. Therefore, a rationale exists for specific policies aimed at this population because patients have varying needs [4, 8, 16, 47]. Future research directions are required as previously indicated [62, 63]. The careful reporting of sex-based differences should become mandatory in research on crack cocaine even if studies do not address it specifically in their objectives [64]. The widespread differences found here in accordance with previous data show that crack cocaine use has a singular impact on each sex, so interventions and policies addressing crack cocaine use likewise require this specificity.
